# HIV in East London: ethnicity, gender and risk. Design and methods

**DOI:** 10.1186/1471-2458-6-150

**Published:** 2006-06-09

**Authors:** Jonathan Elford, Jane Anderson, Cecilia Bukutu, Fowzia Ibrahim

**Affiliations:** 1 City University London, Institute of Health Sciences, St Bartholomew School of Nursing and Midwifery, 24 Chiswell Street, London EC1Y 4TY, UK; 2 Homerton University Hospital NHS Foundation Trust, Centre for the Study of Sexual Health and HIV, Homerton Row, London E9 6SR, UK

## Abstract

**Background:**

While men who have sex with men remain the group at greatest risk of acquiring HIV infection in the UK, the number of new diagnoses among heterosexuals has risen steadily over the last five years. In the UK, three-quarters of heterosexual men and women diagnosed with HIV in 2004 probably acquired their infection in Africa. This changing epidemiological pattern is particularly pronounced in East London because of its ethnically diverse population.

**Design and methods:**

The objective of the study was to examine the social, economic and behavioural characteristics of patients with HIV infection currently receiving treatment and care in hospitals in East London. The research focused on ethnicity, gender, sexuality, education, employment, housing, HIV treatment, stigma, discrimination, religion, migration and sexual risk behaviour. People diagnosed with HIV infection attending outpatient treatment clinics at St Bartholomew's, the Royal London, Whipp's Cross, Homerton, Newham and Barking hospitals (all in East London) over a 4–6 month period were invited to participate in the study in 2004–2005. Those who agreed to participate completed a confidential, self-administered pen-and-paper questionnaire. During the study period, 2680 patients with HIV attended the outpatient clinics in the six participating hospitals, of whom 2299 were eligible for the study and 1687 completed a questionnaire. The response rate was 73% of eligible patients and 63% of all patients attending the clinics during the survey period.

**Discussion:**

A clinic-based study has allowed us to survey nearly 1700 patients with HIV from diverse backgrounds receiving treatment and care in East London. The data collected in this study will provide valuable information for the planning and delivery of appropriate clinical care, social support and health promotion for people living with HIV not only in East London but in other parts of the capital as well as elsewhere in the UK.

## Background

### HIV infection in the UK

There has been a marked shift in the epidemiological pattern of HIV infection in the UK since 1997. While men who have sex with men remain the group at greatest risk of acquiring HIV infection in this country, the number of new diagnoses among heterosexuals has risen steadily year by year [1].

Over 7000 new HIV diagnoses were reported in the UK in 2004. More than half those newly diagnosed with HIV in 2004 had acquired HIV through heterosexual intercourse – three times the number reported five years earlier. In the UK, three-quarters of heterosexual men and women diagnosed with HIV in 2004 probably acquired their infection in Africa. Heterosexual men and women currently account for 48% of all diagnosed patients with HIV receiving treatment and care in the UK [1].

In 2004, sex between men accounted for 2185 new HIV diagnoses in the UK compared with 1370 five years earlier [1]. Increases in high risk sexual behaviour as well as in the incidence of sexually transmitted infections suggest continuing, if not increasing HIV transmission among gay men [2,3]. Gay men remain the group most at risk of acquiring HIV in the UK [1]. They currently account for 43% of all diagnosed patients with HIV receiving treatment and care in this country.

### HIV infection in East London

**Figure 1:**
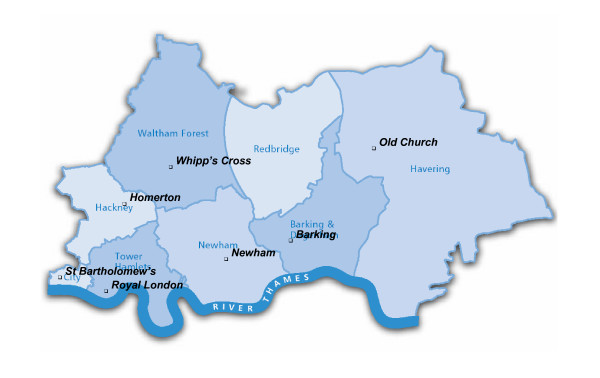
Map of East London with the seven hospitals providing NHS outpatient services for patients with HIV.

National Health Service (NHS) outpatient clinics for HIV in East London are located at seven hospitals: St Bartholomew's, the Royal London, Homerton, Newham, Whipp's Cross, Barking and Old Church (figure 1). In combination, these services make up the North East London Sexual Health and HIV Clinical Network. NHS outpatient clinics provide clinical care to the majority of patients with diagnosed HIV in the UK [1].

According to data collected by the Health Protection Agency (HPA), East London HIV clinics see a greater proportion of black African female patients than clinics elsewhere in the capital [4]. East London patients are also more likely to have acquired HIV through heterosexual transmission rather than through sex between men. For example, the majority of HIV patients treated at Homerton, Newham, Barking and Old Church hospitals are black African (70–80%) compared with less than a third for London as a whole. Half the HIV patients seen at Homerton, Newham, Barking, Whipp's Cross and Old Church hospitals are female compared with only a quarter for London overall. More than three-quarters of the patients at these hospitals acquired HIV through heterosexual intercourse. For London overall this figure is only a third.

### East London population

The HIV treatment services in East London serve the local population to  a greater extent than many other London centres.  At St Bartholomew's at  least half the patients live in East London with the figure rising to  almost 90% for Newham and Homerton hospitals [4].  Historically East  London has attracted migrants and it remains one of the most ethnically diverse parts of the capital. In 2001, depending on the borough, up to two-thirds of the East London population described themselves as belonging to an ethnic minority group. This compares to 40% across London as a whole (personal communication, Marion Mackintosh, GLA).

East London is one of the poorest areas in the capital and indeed of the UK. The expectation of life in East London is one of the lowest in the country with high unemployment rates, low levels of education and poor housing [5,6]. Socioeconomic deprivation impacts on overall health as well as access to and provision of services. For example, out of all the London boroughs Newham has the shortest life expectancy while Hackney has the highest infant mortality rate [7].

### Social and behavioural research among people with HIV in East London

Social and behavioural research into HIV in East London has, for a variety of reasons, lagged behind research conducted elsewhere in the capital. As a consequence, detailed information on the social and economic circumstances of patients with HIV in East London has not been available to date.

We need to better understand the sexual behaviours and attitudes of patients with HIV in the context of their cultural practices and beliefs [8,9]. The relationship between socioeconomic disadvantage, ethnicity and risk also needs to be explored as do the beliefs and practices of men who have sex with men from ethnic minorities [10,11]. More information is required about international patterns of migration among people living with HIV to better understand the relationship between migration, cultural heterogeneity and health [12]. We need to consider beliefs and practices among men and women with HIV infection surrounding pregnancy, termination, and breastfeeding. Further research is also required around uptake of, and barriers to HIV testing and the stage at which people are diagnosed with HIV paying particular attention to socioeconomic and cultural factors [13].

To address the gaps in our knowledge we undertook research funded by the St Bartholomew and Royal London Charitable Foundation with additional support from City University London (Institute of Health Sciences, St Bartholomew School of Nursing and Midwifery). This research, known as the East London project, has been conducted by researchers at City University London and Homerton University Hospital, in close collaboration with NHS HIV treatment centres across East London.

### East London project

The East London project has been designed to address the core research question: What are the social, economic and behavioural characteristics of patients with HIV infection currently receiving treatment and care in East London?

The East London project focuses on ethnicity, gender, sexuality, socioeconomic deprivation, education, employment, housing, religious affiliation, use of services, disclosure, discrimination, migration, HIV testing, diagnosis and treatment, pregnancy and sexual risk behaviour. The data collected in this study will provide valuable information for the planning and delivery of appropriate clinical care, social support and health promotion for patients with HIV in East London and, hopefully, elsewhere in the UK.

## Design and methods

### Target population

The target population comprised all patients diagnosed with HIV infection receiving treatment and care in East London hospitals (i.e. at St Bartholomew's, the Royal London, Homerton, Newham, Whipps Cross, Barking and Old Church). The majority of patients with diagnosed HIV infection in the UK seek clinical care in NHS outpatient clinics [1]. Consequently, an NHS clinic sample is broadly representative of all those living with diagnosed HIV.

In 2003, at the planning stage of the study, HPA data indicated that just over 2500 patients with HIV were receiving treatment and care in East London clinics (St Bartholomew's 536; Royal London 807; Whipp's Cross 197; Homerton 276; Newham 433; Barking 209; Old Church 51) [4]. Taking into account new diagnoses, it was anticipated that approximately 2800–3000 patients would be attending HIV clinics in East London when the study was actually conducted (2004–2005).

### Recruitment

Based on previous research among people living with HIV in London we estimated that we would need to recruit a total of 1000 patients from the East London clinics overall [2,10,12,14,15]. This would provide sufficient power at a 5% level of significance to compare the key variables of interest according to gender (male, female), ethnicity (white, black African) and sexual orientation (gay/bisexual, heterosexual).

People diagnosed with HIV infection attending NHS outpatient (HIV) clinics in the East London hospitals over a 4–6 month period in 2004–2005 were invited to participate in the study. Patients with a limited command of English were ineligible for the study as were those who were too ill or too distressed to complete a questionnaire. In some clinics eligible patients were approached by a trained member of the research team in the waiting area. In other clinics a doctor or nurse first mentioned the research project to the patient during a routine consultation. If the patient expressed interest in the study they then talked to a trained member of the research team in the clinic. Some patients were only in the clinic for a short time so there wasn't an opportunity to invite them to take part in the study.

All patients who expressed interest in the project were provided with written information about the research, contact details of the research team as well as helpline numbers. The member of the research team in the clinic explained why the study was taking place, what it entailed, what the anticipated benefits would be and answered any queries about the study. Patients were then invited to participate. If the patient agreed to participate having read the information sheet, they were asked to sign a consent form. Patients were only enrolled after they have given fully informed consent in writing. No financial remuneration was offered to participants.

Study participants were asked to complete a self-administered pen-and-paper questionnaire in the clinic and return it in a sealed envelope to the team member (further information about the questionnaire in Data collection below). Those who did not have sufficient time to complete the questionnaire in the clinic took it home with them and mailed it to the research team in a pre-paid envelope. All patients were asked to complete only one questionnaire.

In each hospital we distributed questionnaires over a 4-6 month period.  In this way we included not only frequent clinic attenders but also  those whose HIV infection was stable and who only attended once every  two to three months.  A trained member of the research team was present  in the clinic on all the days that recruitment took place to liaise with  clinic staff and patients.  For logistic reasons we introduced  questionnaire distribution into the clinics in stages starting with the  outpatient clinic at Homerton (May 2004) followed by St Bartholomew's  (June 2004), the Royal London (June 2004), Newham (October 2004), Whipps Cross (October 2004) and finally Barking (February 2005). For administrative reasons, it was not possible to include Old Church in the study but this was the smallest clinic accounting for only 2% of all patients receiving treatment and care in East London [4].

Over the study period, 2680 individual patients attended the outpatient clinics in the six participating hospitals of whom 2299 were eligible for the study (table 1). Of those who were eligible, 1687 completed and returned a questionnaire exceeding the original estimate of 1000 participants. The response rate was 73% of eligible patients and 63% of all patients who attended the clinics during the survey period (1687/2680). The response rate varied by clinic (from 64%-88% of eligible patients). The number of patients attending the outpatient clinic in each hospital during the 4–6 month survey period reflected the number reported by the HPA in its annual survey of HIV patients receiving treatment and care in England and Wales (SOPHID) (personal communication, Sean Overett, December 2005). There were marked differences between hospital clinics in the ethnicity, gender and sexual orientation of the respondents (data available from authors on request). Similar differences between East London clinics have been reported by the HPA in its annual Survey of Prevalent HIV Infections Diagnosed (SOPHID) [4] (personal communication, Tim Chadborn, March 2006). The similarity of our dataset and the SOPHID dataset provides evidence that our sample reflects the diversity of HIV patients receiving treatment and care across East London.

Of the 1687 respondents, 758 were gay/bisexual men (646 white, 112 ethnic minority), and 704 were black African heterosexual men and women (480 women, 224 men); together they account for nearly 90% of the HIV patients in our sample. Gay men and black African heterosexual men and women are the groups most affected by HIV in the UK currently [1].

### Data collection

Information was collected by means of self-administered pen-and-paper questionnaires distributed in the HIV outpatient clinics. Each participant was allocated a unique study number. Questionnaires were confidential and anonymous; they contained no information that would allow an individual respondent to be identified. A separate secure register matching study number to clinic number, stored away from the questionnaire data, was maintained by the investigators.

The questionnaires sought detailed information on socio-demographic characteristics (age, sex, ethnicity, education); socio-economic circumstances (housing, employment, income, family); religion; country of birth and migration; HIV testing, diagnosis and treatment; disclosure of HIV status; discrimination; reproductive history and children; anxiety and depression; sexual behaviour with main and casual partners; beliefs and attitudes concerning HIV; use of community and support services; use of the Internet.

The questionnaire for the most part contained closed questions but there were some open questions as well. Standardised and validated questions were used where possible drawing on research already conducted in the UK (e.g. by Sigma Research, UCL, Mayisha, Padare, Shibah and City University) [2,3,10,14,16-20] and elsewhere (e.g. in USA) [21,22]. Copies of the questionnaire are available from JE.

The questionnaire was piloted extensively at the development stage of the study and revised in the light of feedback and comments to ensure it was suitable for a diverse sample of people living with HIV comprising ethnic minority heterosexual men and women as well as gay men. The questionnaire was in English. The possibility of translating the questionnaire into other languages was considered but because of the large number of languages spoken in the clinics this proved not to be feasible. In fact language difficulties accounted for only a small number of exclusions from the study (71 people; 3% of all those seen by a member of the research team) (table 1)

### Ethics

The research protocol was approved by the East London and The City Local Research Ethics Committee, the Redbridge and Waltham Forest Local Research Ethics Committee and City University London Research Ethics Committee.

### Advisory group

An advisory group was set up at the start of the project to provide guidance and support. The advisory group was made up of representatives from the African HIV Policy Network, Terrence Higgins Trust, Health Protection Agency, Positively Women, Organisation of Positive African Men, North East London HIV Commissioning Team, MRC Social and Public Health Sciences Unit and St Bartholomew School of Nursing and Midwifery.

## Discussion

A clinic-based study has allowed us to survey nearly 1700 patients with diagnosed HIV in East London. The diverse nature of the sample reflects the changing epidemiological pattern of HIV in the UK which is particularly pronounced in East London because of the heterogeneity of its local population. A strength of the study is that a clinic sample is broadly representative of all those living with diagnosed HIV as the majority of those diagnosed HIV positive in the UK access treatment and care within the NHS.

The findings from this questionnaire study will allow us to:

• Describe in detail the socioeconomic and behavioural characteristics of patients with HIV in East London

• Provide data to both providers and users of health care to improve clinical and social support for patients with HIV treated in East London

While the study is geographically defined, it is likely that the findings will have relevance for HIV patients in other parts of London and the UK. Hopefully our findings will provide a foundation for developing research and interventions nationally among people living with HIV.

## Competing interests

The author(s) declare that they have no competing interests.

## Authors' contributions

JE and JA conceived the study; JE, JA and CB participated in its design; CB was responsible for questionnaire distribution, data collection and data entry; FI was responsible for data management and analysis; JE drafted the manuscript with input from JA and FI. All authors read, revised and approved the final manuscript.

## Pre-publication history

The pre-publication history for this paper can be accessed here:



## Figures and Tables

**Table 1:** Number of people attending the HIV outpatient clinics and participation in the East London project, 2004–2005

Hospital		Homerton		St Bartholomew's		Royal London		Newham		Whipp's Cross		Barking		Total	
*Data collection from:*		*26 May 04*		*16 Jun 04*		*29 Jun 04*		*13 Oct 04*		*29 Oct 04*		*10 Feb 05*		*26 May 04*	
*Data collection till:*		*05 Nov 04*		*10 Nov 04*		*23 Dec 04*		*17 Feb 05*		*25 Feb 05*		*16 June 05*		*16 June 05*	
Patients attending the clinic		343		485		946		449		226		231		2680	
Not seen by the researcher		67		41		31		42		30		21		232	
Not eligible for the study		37		18		16		53		16		9		149	
*Reason*	*Too ill*	*13*		*14*		*2*		*4*		*5*		*4*		*42*	
	*Language*	*19*		*2*		*9*		*30*		*9*		*2*		*71*	
	*Newly diagnosed*	*5*		*2*		*5*		*19*		*2*		*3*		*36*	
Eligible for the study & invited to participate		239	*100%*	426	*100%*	899	*100%*	354	*100%*	180	*100%*	201	*100%*	2299	*100%*
Declined to participate		35	*15%*	26	*6%*	48	*5%*	31	*9%*	17	*9%*	14	*7%*	171	*8%*
Took questionnaire away		50	*21%*	88	*21%*	157	*18%*	95	*27%*	40	*22%*	11	*5%*	441	*19%*
but didn't return it															
Completed a questionnaire		154	*64%*	312	*73%*	694	*77%*	228	*64%*	123	*69%*	176	*88%*	1687	*73%*
	*Completed it in the clinic*	*107*		*247*		*514*		*189*		*102*		*162*		*1321*	
	*Returned it by post*	*47*		*65*		*180*		*39*		*21*		*14*		*366*	
